# The Internet of Things in Geriatric Healthcare

**DOI:** 10.1155/2021/6611366

**Published:** 2021-07-17

**Authors:** Deblu Sahu, Bikash Pradhan, Anwesha Khasnobish, Sarika Verma, Doman Kim, Kunal Pal

**Affiliations:** ^1^Department of Biotechnology and Medical Engineering, National Institute of Technology, Rourkela 769008, India; ^2^TCS Research and Innovation, Kolkata 700156, India; ^3^Council of Scientific and Industrial Research-Advanced Materials and Processes Research Institute (CSIR-AMPRI), Madhya Pradesh, Bhopal, India; ^4^Department of International Agricultural Technology and Institute of Green Bioscience and Technology, Seoul National University, Seoul, Republic of Korea

## Abstract

There is a significant increase in the geriatric population across the globe. With the increase in the number of geriatric people and their associated health issues, the need for larger healthcare resources is inevitable. Because of this, healthcare service-providing industries are facing a severe challenge. However, technological advancement in recent years has enabled researchers to develop intelligent devices to deal with the scarcity of healthcare resources. In this regard, the Internet of things (IoT) technology has been a boon for healthcare services industries. It not only allows the monitoring of the health parameters of geriatric patients from a remote location but also lets them live an independent life in a cost-efficient way. The current paper provides up-to-date comprehensive knowledge of IoT-based technologies for geriatric healthcare applications. The study also discusses the current trends, issues, challenges, and future scope of research in the area of geriatric healthcare using IoT technology. Information provided in this paper will be helpful to develop futuristic solutions and provide efficient cost-effective healthcare services to the needy.

## 1. Introduction

The rapid advancements in clinical science and technologies have significantly increased the average life expectancy of humans across the globe [1]. This led to a substantial rise in the geriatric population. In 2015, the number of geriatric people was nearly equal to 8.5% of the world population and it was estimated that it will increase to 12% and 16.7% by the years 2030 and 2050, respectively [2]. As compared to other age groups, elderly persons are more prone to several health-related issues such as diabetes, hypertension, asthma, and chronic diseases. Hence, the elderly group of people needs the utmost attention in terms of medication, treatment, and care especially if they chose to live an independent life. The prime constraint in availing of a good healthcare service is its rising cost [3]. Also, the aged group cannot be physically present at the health center each time they face a health issue. The reason may be either the increased cost or the unavailability of a good health center with all advanced technologies. This has inspired various research communities to go for other alternatives that can reduce the expenditure while delivering quality healthcare services to the patients. With the wide use of advanced technologies and Internet services, and sensors, it is now possible to avail a range of healthcare services at home. This allows the geriatric people to lead an independent life while receiving standard clinical service at home.

**Figure 1:**
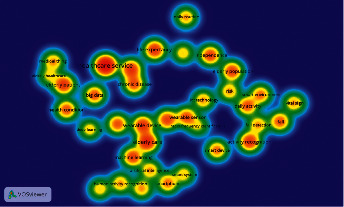
Density plot showing the evolution of trending keywords on the application of IoT in geriatric care. The plot was generated using VOSviewer software.

In the last decade, several state-of-the-art technologies such as machine learning, deep learning, the Internet of things (IoT), data analytics, and artificial intelligence have opened up a new arena of research in the field of healthcare services. In particular, IoT has shown substantial growth in recent years and is expected to continue even in the future [4]. IoT is defined as a network of physical objects with embedded technology that can sense and interact with the surrounding environment and provide autonomous communication [5]. It is a platform for seamless and better integration between computer-based systems and the physical world. This has enabled humans to connect with anyone, anywhere and anytime [6]. IoT has taken maximum use of real-time monitoring technology to provide efficient healthcare services [3]. The description of the basic architecture of an IoT-based healthcare system is provided in supplementary section S1. The application of IoT technologies in healthcare systems reduced the repeated manual examination of various physiological parameters such as body temperature, blood pressure, blood oxy gen levels, and heart rate. Further, these systems provide an automatic and precise collection of health information that could potentially speed up the process of treatment, lower the cost of hospitalization, and enhance the user experience [7]. This helps to build digital health records that can be accessed from a distance/remote location. Though numerous IoT-based systems such as smart homes, smartwatches, and many more have been developed in recent years, smart wearables are widely used as they provide the advantage of continuous monitoring of the physiological parameters without discomforting the patients [8]. These devices also provide a platform that can connect patients with healthcare providers through the Internet. Furthermore, such devices allow the collection of a huge amount of data and help to develop more precise clinical guidelines [9]. These data sources can also be used to develop automated IoT devices for real-time monitoring [10]. The IoT technology is efficiently used to provide rehabilitation, diagnosis, and healthcare monitoring [11]. However, to track the evolution and recent trends of the ongoing research in the field of IoT and elderly care, we performed a bibliometric analysis (using the software VOSviewer) on the collected articles from the “Web of Science” database with a keyword search {“Internet of Things” AND (“Elderly” OR “healthcare”) AND “Elderly care”}. The result showed 184 articles. Using these articles, we have analyzed the evolution of important keywords used in the aforementioned field over time (Figure 1) as well as their correlation. The density plot shows that “healthcare services,” “elderly patients,” “elderly care,” and “wearable devices” are some of the important keywords in the previously published literature. A cluster of keywords, namely, “medical things,” “elderly healthcare,” “elderly patients,” and “health conditions,” were found to represent the association between the Internet of medical things and elderly healthcare. Unfortunately, the cluster shows a weaker density that represents an insignificant growth over time. This may be due to the existence of limited research in the field of geriatric care. Further, only a few articles have discussed the issues and challenges in employing the IoT-based technology in providing the elderly healthcare services. Hence, there is a need for a detailed review in the aforementioned field of study.

Consequently, this paper aims to provide comprehensive information emphasizing the recent or possible application of IoT technology in geriatric care services. The outcome of this research could be beneficial for researchers, especially the IT professionals who want to use the technology in various healthcare applications. The current paper discusses the need for health monitoring in the geriatric population, current sectors that are getting influenced by the IoT technology, the applications in various healthcare services, present issues, and future challenges. The knowledge shared in this research will help the researchers/scientists to develop technologies that can deliver healthcare services to the needy, promote service delivery to the elderly, and increase their quality of life.

## 2. Need for Health Monitoring in the Geriatric Population

Aging is a normal biological process that leads to the occurrence of age-related diseases. Such diseases are caused due to either endogenous or exogenous factors. This results in functional and morphological changes in all organs and systems [12]. The physiological changes may result in the precipitation of chronic diseases like osteoporosis and depression. Quite often, such diseases are hidden and remain atypical for a longer duration. In the case of the geriatric population, the diseases progress at a faster rate and may get complicated easily. The treatment of geriatric-related diseases results in polypharmacy. Unlike in the middle-aged and young adults, the diagnosis and treatment modality in the geriatric population generally requires a different approach. However, many a time, after receiving suitable treatment and diagnosis, the geriatric patients become weak both mentally and physically. Hence, rehabilitation of the geriatric population has been regarded as essential for a healthy society. The rehabilitation process helps the healthcare givers to maintain optimum health and quality of life for geriatric patients. Though the list of health issues in older age is large, some of the most common issues are discussed in the current section.

### 2.1. Auditory and Visual Impairment

Visual and hearing impairments restrict the patient's ability to express clearly and interact with the immediate environment. Hearing loss can result in depression, irritability, frustration, isolation, loneliness, cognitive impairment, and compromised physical mobility [13]. The solution to auditory impairment empowers one's daily routine work and psychological behavior. This shows a positive effect in various areas like a family relationship, enjoyment of leisure activities, telephone communication (emergencies and social touch), and communicating the health issues and daily needs to others. This empowers them to live safely and independently. Along with auditory, there is also a gradual deterioration of visual sensory function with aging. As compared to hearing impairment, visual impairment exerts a deep impact on the health status of the geriatric population. Vision loss is associated with high-risk consequences like increased social isolation, reduced self-image, depression, and physical disability [14]. The physical disability includes a lack of ability to perform daily activities, mobility, and reduced participation in other activities. The elderly population with both visual and auditory impairment undergo two times more difficulties than the single-impairment population. They face difficulties in managing medication regimens, ambulation, and performing their daily activities [15].

### 2.2. Falls

Every year, 30% of the geriatric population aged 65 years and older suffer from falls. About 14–50% of people who encounter falls are unable to rise after it [16]. In most cases, they are found lying on the ground and are later discovered by others. Moreover, the women population experience a greater proportion of falls. This observation can be due to the fact that women constitute a majority of the total population due to the higher mortality rate of the elderly men population [17]. Geriatric patients with gait dysfunction have a high risk of falls. The risks may include traumatic brain injury, fractures, and joint dislocation. Patients associated with falls undergo pain, loss of stamina, and declined function. Around 40% of people sustaining falls had reported continued pain and hence have been prescribed bed rest for a few months after the fall. Suffering a fall can damage self-confidence and self-trust and jeopardize the independence of a geriatric patient as it is associated with long-term treatment and care [18].

### 2.3. Osteoporosis

Osteoporosis is extremely prevalent in old age. It affects nearly one-half of the population that is aged above 75 years and one-third of the postmenopausal women population [19]. It may result in bone fracture even with minimal trauma. Around the globe, millions of elderly people suffer from osteoporosis that commonly leads to vertebral, hip, or wrist fractures [20]. Around 25% of women who are aged 50 years and older have one or more vertebral compression fractures that can be related to osteoporosis. Unlike falls, patients with osteoporosis undergo long-term hospitalization and have to receive medication for a long time. This can result in compromised physical mobility, irritability, frustration, and damage to the self-esteem of the patients.

### 2.4. Malnutrition

Malnutrition occurs when there is a deficiency of vitamins, minerals, protein, and other essential substances that the human body needs for its proper functioning [21]. Additionally, various psychological factors such as loneliness, depressive symptoms, bereavement, and cognitive decline also contribute to malnutrition in elderly persons [22]. A poor nutritional status may also be found in old-aged people who rely on others for meals. Poor nutrition directly affects the patient's medical condition and functional status. This can decrease one's immunity and results in poor recovery from diseases.

### 2.5. Depression

Depression is one kind of mental health disorder, which has been diagnosed among most of the geriatric population. The disease shows symptoms of fatigue, sleep disturbances, and weight loss. It has quite often been associated with coexisting medical conditions. Various medical conditions like Alzheimer's disease, Parkinson's disease, malnutrition, cancer, stork, HIV infection, hypothyroidism, hyperparathyroidism, hepatitis, arthritis, skin problems, and speech disorders may lead to depression [23]. In the geriatric population, depression can decrease walking speed, standing balance, ability to rise from a chair, and functions that are associated with daily activities [24]. Moreover, it contributes to the individual's disability, functional failure, and losing hope to live a quality life. Patients with depression usually need prolonged treatment and diagnosis. Proper diagnosis and care of depression can significantly decrease further disability, restore functions, and help to maintain a healthy life in the geriatric population.

### 2.6. Delirium and Dementia

Cognitive impairment, such as delirium and dementia, is quite common in the elderly population. Generally, delirium is caused due to severe or chronic illness, changes in metabolic balance, medication, and infection [25]. It is a disorder of attention that affects mental, sensory, behavioral, and emotional functioning. This disease is usually acute and temporary. Around 40% of delirium patients face hallucinations that get worse at nighttime. The symptoms of delirium in the geriatric population may last for hours to weeks [26]. However, dementia is not a part of aging. It is a cognitive impairment that resulted in the loss of memory, thinking ability, and other mental abilities. Patients with dementia have short-term memory problems. They tend to forget the events quickly and hence have been found to repeatedly ask the same question. This disease in the geriatric population disrupts their daily activities and leads to social impairment.

## 3. Influence of IoT on Geriatric Health Monitoring

Healthcare technologies that employ IoT, artificial intelligence, cloud computing, and mobile computing are crucial in the development of an efficient medical system that can facilitate a better life for the aging population. These technologies are used to design portable devices such as wearable devices and sensors, smartphones, and rehabilitative devices. Such devices promote public healthcare services at remote locations. The efficient use of these techniques helps to educate customers/patients, reduces the medical load of the health centers and doctors, and improves real-time monitoring. These devices have significantly influenced geriatric healthcare monitoring. The healthcare monitoring domains can be categorized based on the services they provide to the patients. Some of the most widely influenced areas are discussed in the subsequent section.

### 3.1. Wearable Devices and Sensors

**Figure 2:**
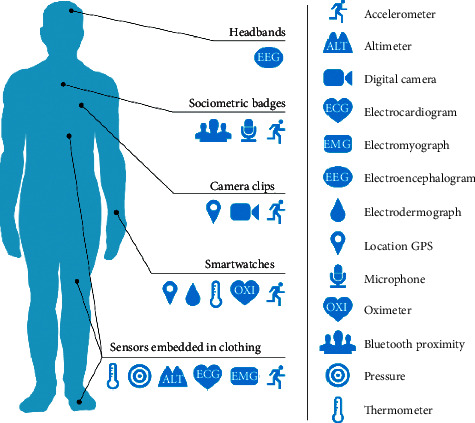
Example of different consumer wearable sensors and devices (reproduced from [32]).

Wearable devices and sensors are the measuring tools that are used for real-time monitoring. These devices collect physiological information from the human body and can also monitor physiological activities. In elderly people, the application of wearable sensors has diverse uses such as fall detection [27], sleep pattern monitoring [28], cardiac health monitoring [29], and sedentary behavior [30]. The wearable devices are capable enough to alert the patients and healthcare providers when an adverse situation arises. Real-time monitoring can be achieved through a continuous recording, storing, and upgradation of the healthcare data at the cloud server. Due to the advantage of easy handling and affordability, these devices have become popular in the recent years. Seneviratne et al. have discussed various commercially available wrist-worn devices in the market such as Samsung Gear S2, Empatica, Apple iWatch, Fitbit Flex, Pebble Time and other accessories such as smart jewelry, skin patches, and e-textiles. The authors further reported the challenges associated with these commercial wearables which include security threats and confidentiality of information [31]. In another study, the consumer wearable sensors such as headbands, camera clips, smartwatches, and various embedded sensors in clothing have been explored (Figure 2). These devices give direct access to the patients to analyze their healthcare data and contribute to their better health [32]. Kekade et al. had performed a survey to evaluate the usefulness of commercially available devices. The findings of this study proposed that more than 60% of elderly people showed interest in the future use of wearable devices. This inferred the positive and significant growth of this technology in the future.

### 3.2. Ambient Assisted Living

Ambient assisted living- (AAL-) based medical devices/systems use the information and communication technologies to transform the lives of elderly people and other patients by providing them an independent life [33, 34]. These devices integrate into the home and life of patients in an intelligent and prevalent way. This further increases the quality of life and significantly reduces healthcare expenses. The AAL-based devices mainly focus on monitoring the patient's daily activities, detect health abnormalities, and deal with emergency conditions. Various AAL-based systems have been reported in the literature which help in achieving the aforementioned goal in elderly people. Dohr et al. had introduced the “Keep In Touch (KIT)” technology in AAL, which combined smart objects and technologies such as near-field communication (NFC) and radiofrequency identification (RFID) to efficiently process healthcare information obtained from the patient's body [35]. The KIT technology also provides a communication channel for the sharing of information among geriatric patients, healthcare providers, and doctors. In [36], the AAL system captured user information through various sensors and detected the activity of daily living (ADL). Herein, the authors have tried to correlate the probability of calculating various illnesses with the environmental scenario (season, air quality, the intensity of sunray, etc.). Loza-Matovelle et al. had developed an AAL system by integrating the robotic technology with another network sensor to provide assistant service for elderly people [37]. Smart homes [38], wearable sensors [39], and mobile technologies [40] have also been integrated with the AAL technologies to provide remote monitoring with a functional support system.

### 3.3. Telemedicine

A telemedicine system provides medical care through establishing audiovisual communication between doctors and patients. The term “medical care” involves diagnosis, treatment, consultation, and prescription of medicines [41]. Provision of secure and safe medical care involves the use of mobiles and communication technologies, home sensors, wearable sensors, and so forth. In [42], a telemedicine system has been reported, which can monitor and record physiological signals such as heart rate, blood flow, and myoelectric signals. The recorded data could be accessed through a mobile phone or tablet and further processed and analyzed. Stradolini et al. have developed a cloud-based telemedicine system for anesthesia monitoring. The system allows the anesthesiologist to closely monitor the sedated patients through an android app [43]. In [44], a smart healthcare service model has been designed and proposed. The system provides an authorized telemedicine infrastructure for geriatric patients where the healthcare professional can continuously monitor the activity of the caregiver and patients and also can interact with the patients.

### 3.4. Mobile Healthcare Services

**Figure 3:**
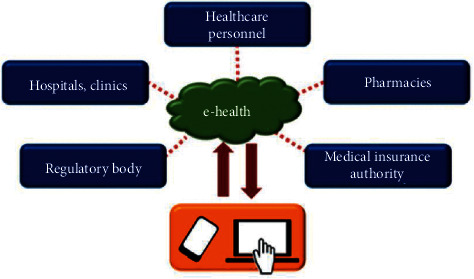
A mobile IoT environment (reproduced from [45]).

The mobile healthcare system helps in preserving a patient's vital information in the form of electronic medical reports and allows healthcare professionals to access this information when needed. Most of the mobile healthcare systems use the cloud server to store the information and use a mobile app as an interface to connect patients, caregivers, and doctors. This process enhances the accessibility of medical information and the efficiency of the system. It has been reported that older adults mostly prefer a mobile phone over other electronic devices such as computers. The portability of these devices and the ease of accessibility may be the reason behind their huge popularity. In many IoT-based systems, the smartphone is used as a gateway for data communication among hospitals, pharmacies, medical authorities, and patients (Figure 3). Saraubon et al. have proposed a smart geriatric care system using IoT and mobile technology to detect falls, monitor heart rate, and provide real-time video monitoring [46]. In another study [47], a data mining-based approach was employed in the mobile healthcare system for activity recognition. Some of the other explored areas where efficient use of mobile technology has been employed for geriatric care include chronic diseases, cardiac diseases, diabetes, and mood [48–50]. The use of mobile devices also allows elderly people to share their health-related information with their families.

### 3.5. Robotic Technology

**Figure 4:**
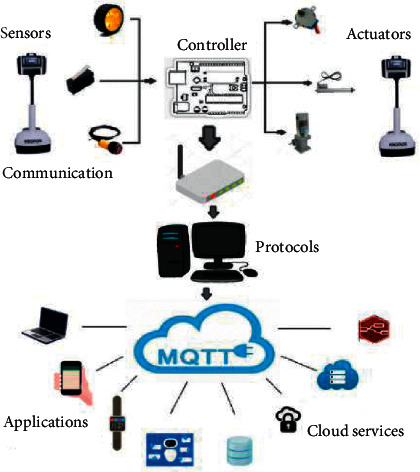
Components of an IoT-based healthcare system integrated with robotic technology (reproduced from [37]).

Robotic technology has transformed elderly healthcare by taking into consideration the potential use of human-automation interaction. This technology assists the geriatric population in their daily activity, alerts the patients about their health issues, ensures the safety of the patient, and provides social support. Bogue has mentioned three different types of robots that could be employed in geriatric care. These include household robots, companion robots, and assistive robots [51]. The integration of robots in IoT-based healthcare systems helps elderly disabled persons to perform physical tasks. The components of an IoT-based system with robotic technology are represented in Figure 4. It is possible to create a sense of the physical presence of the caregivers and doctors from a distance that can virtually interact with the patients through a robotic body [52, 53]. The literature also reported the efficient use of telepresence robotic technology in monitoring ECG, video conferencing, and reminding patients to take medicine [52]. In [54], IoT, wireless communication, and automation technologies were integrated into a wheelchair for real-time monitoring. The system could visualize the surrounding environment with the help of an array of cameras. This helped the system to achieve safer navigation tasks. In a recent study, researchers have combined robotics with other sensing technologies in an IoT-based AAL environment to provide a higher human-robot interaction. Such systems can understand the patient's needs and can act accordingly in a more adaptive manner [37].

## 4. IoT Applications in Geriatric Care

IoT has transformed the healthcare industry with advanced sensing technologies, communication protocols, and data analytics techniques. This has influenced the life of geriatric people by improving their quality of life and providing a wide range of healthcare applications. In the subsequent sections, some of the most prominent applications of the IoT in the healthcare industry with special attention to geriatric care are discussed.

### 4.1. Monitoring Clinical Health Parameters

The clinical health parameters (e.g., blood pressure, pulse rate, temperature, oxygen saturation, blood glucose, balance, gait, and lipid profile) act as vital signs for various diseases and health abnormalities. In the case of the elderly, early attempts must be made to regularly obtain the health status. This would help in the timely diagnosis of a disease and avoid sudden complicacy. Hence, continuous observations of these parameters are needed. Several studies [55–58] in the past have addressed the role of IoT technology for monitoring different health parameters.

Blood pressure fluctuation is one of the most common diagnostic parameters for measuring several health issues. The use of a conventional device requires a helping hand for the measurement of blood pressure. These issues could be eliminated through the integration of IoT technology that brings about more independence to the patients. Anh Dinh et al. designed a device that makes use of the electrocardiogram (ECG) and the photoplethysmogram signal from the fingers to obtain blood pressure from the fingertip [59]. In [60], a device for obtaining both systolic blood pressure and diastolic blood pressure separately has been proposed. The results showed a higher confidence interval than that of the results obtained from the oscillometric method. Body temperature is a vital sign in many health issues. The inaccuracy in measuring this parameter may lead to failure in identifying patient complications and adversely influence the diagnosis process [61]. The IoT-based devices enable real-time monitoring of temperature from a distance. In [62], a single-chip computer (Raspberry Pi) board was used to collect and process data from the various sensors. Further, the recorded information was displayed using a monitor.

Aging people are highly prone to diabetes. Continuous monitoring of the glucose levels helps the physicians to provide medication at right time. One of the possible solutions to avoid a repeated visit to the hospital is the IoT-enabled remote monitoring technology. In [63], a noninvasive method named “Gloco” has been proposed. This method used an infrared LED in the fingertip and calculated the blood glucose level based on received light intensity. The glucose level reading was then sent to a mobile phone where the recorded data could be accessed through a mobile application. In another research [64], a smart device was developed, which used a single-chip computer (Raspberry Pi), a power bank, a visible laser beam, and a Pi camera, all integrated into a hand glove. The glove provides the body glucose level information. The generated data then processed by an artificial neural network and the results could be viewed on any smartphone.

In recent studies, researchers have focused on measuring multiple health parameters at the same time. Kumar et al. have proposed a system that monitors pulse rate, respiratory rate, and body temperature using noninvasive sensors. The system used a microcontroller to process the sensor data and display the same using the “ThingSpeak android application” [65]. In another study [66], a remote health monitoring system has been proposed, which used a single-chip computer (e.g., Raspberry Pi) and IoT for measuring the body temperature and pulse rate concurrently. Along with these two measures, the device is also capable of recording other health parameters including blood pressure, heart rate, and respiration.

Most of the conventional devices, which measure the important health parameters, are mostly used in the intensive care unit (ICU) and operation theaters. However, they still have not achieved a high acceptance by common people. This is due to its higher cost. In the future, IoT devices must be designed at a lower cost with the use of cheaper sensors and materials. Currently, conventional devices are transforming into electronic devices that are user-friendly and need less human intervention. For example, the use of infrared (IR) technology in measuring body temperature is more popular [67]. The infrared thermometer measures the body temperature from the forehead using the IR sensor without any physical contact with the patient. The method not only reduces the risk of cross-contamination but also prevents the spread of contagious diseases including COVID-19. This type of technological intervention allows reducing the use of mercury, which is used in conventional thermometers [68]. The main challenge associated with the measurement of the blood glucose level is that it is unstable and depends on the food intake status of a person. As per the National Institute for Health and Care Excellence (NICE), one of the possible indicators for the measurement of blood glucose level can be HbA1c (glycated hemoglobin). It has also been experimentally found that an average increase of 2 mmol/L in the blood glucose results in the rise of the HbA1c level by 11 mmol/L [9]. Sensing and measuring HbA1c is still a potential challenge and is being explored as a measurement tool by many researchers.

### 4.2. Activity Recognition

**Figure 5:**
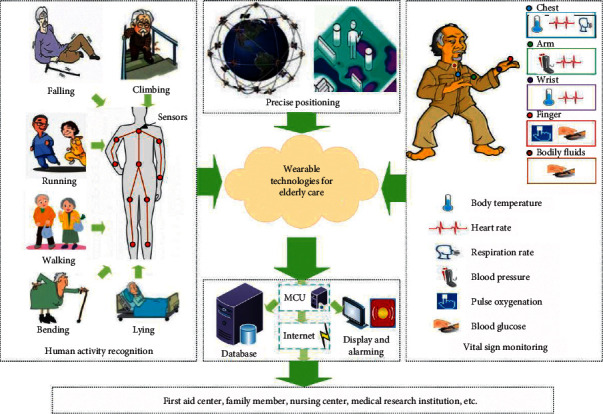
Schematic functions of elderly care including real-time monitoring of indoor positioning, physical activities tracking, and vital signs (reproduced from [73]).

The real-time monitoring of human activity is a useful and efficient tool in elderly care as it is associated with maintaining daily activities, health monitoring, and enhancing the safety and security of elderly people. Though fall detection of the user is the prime focus, the recognition of different activities can also be employed to analyze the user behavior. As per the data given by the World Health Organization, more than 28% of the aged population gets affected by falls each year [69]. It is also expected that a lack of preventive measures may double this value by 2030. A fall event may not only cause physical injuries but also have psychological consequences including anxiety, depression, and fear of falling. The IoT devices that are capable of tracking human activity can contribute to reducing this kind of adverse event. This can be achieved by employing advanced algorithms and various body-worn sensors in an IoT environment [70]. Numerous studies [71–73] in the past have investigated the potential of IoT in delivering different solutions to activity recognition and fall detection. The authors in [74] reviewed various state-of-the-art wearable technologies in geriatric care for activity recognition, position monitoring, and vital sign monitoring (Figure 5). They investigated the use of various sensors and their integration in monitoring positions and activities. The authors have also discussed the future aspects of developing a “smart clothing” system. In [75], a framework for IoT-enabled Personalized Intelligent Assistant has been proposed for helping geriatric patients in performing their daily activities. The system also analyzes the daily activities and reports if any abnormality in behavior was observed. Arifoglu and Bouchachia have developed a wearable device that used a 3D-axis accelerometer embedded with a 6LoWPAN for falls detection in elderly people [76]. The sensor data was processed using a tree-based big data model that functioned in an IoT gateway. If falls were detected, an emergency alert was activated to notify the caregivers. Further, it generates an emergency alert to the family members or caregivers when a fall was detected. The IoT-based systems have employed various machine learning algorithms for detecting and classifying multiple activities. Arifoglu and Bouchachia have proposed a similar method for geriatric patients with dementia. Three variants of Recurrent Neural Network have been used for the detection of abnormal behavior and activity [76]. In [77], the authors have employed various wearable sensors and machine learning models for monitoring activities of geriatric patients with Parkinson's disease. Herein, gyroscopes and triaxial accelerometers have been used for sensing these activities. Additionally, machine learning algorithms were used for recognizing these activities. In [77], a fall detection cum emergency response system has been presented. The system employed deep sensors to get binary images of the elderly persons who were tracked by Microsoft Kinect SDK. The features of the binary image were extracted using the histogram of oriented gradient (HOG) method. The status of the fall was evaluated using the support vector machine (SVM) algorithm.

Integration of IoT for activity/fall detection brings about many advantages and has been widely used in terms of remote access. However, some major challenges need to be addressed in the future. The use of wearable sensors in these systems questions their real-time use. This can be explained by the fact that it is not possible to wear a sensing device all the time. Moreover, the use of these wearable sensors also has its inherent flaws. This includes the loss of wearable devices, maintenance burden, discomfort in wearing, and lower battery life [77]. Hence, device-free systems must be developed in the future with minimal use of wearable sensors. The use of multiple sensors and different communication modules used for the detection of multiple activities increases the computational complexity and processing time. Another major challenge for such types of devices is identifying the human poses and detecting a change in a pose while detecting a fall event. In a recent study, this issue has been eliminated with the applications of convolutional neural network algorithms [78]. The authors have used a series of poses to differentiate between a fall event and a nonfall event. In [79], the authors proposed a method that used three-axis accelerometer and gyroscope to detect fall in elderly person by differentiating static position from dynamic position. The device also provided information about the four kinds of positions, falling backward, falling front, jumping, and sitting fastly, by considering the velocity and acceleration of patients. Although these systems are incorporated with many advanced features, they lack in achieving higher functionality and customer demands.

### 4.3. Chronic Diseases Monitoring

**Figure 6:**
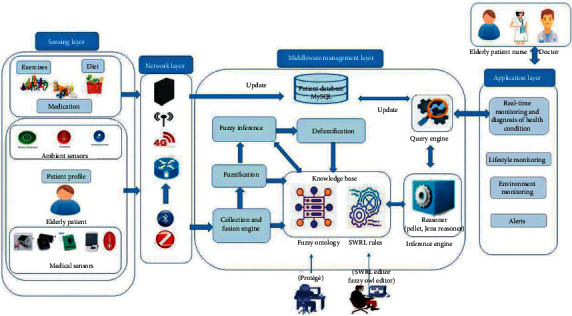
Basic architecture of fuzzy ontology-based healthcare system (reproduced from [87]).

Chronic diseases are one of the leading causes of death in elderly people [79]. As per a medical survey [80], about 80% of geriatric people aged above 65 suffer from at least one chronic disease. The most common chronic illnesses associated with elderly people include diabetes, cardiovascular diseases, cancer, depression, Alzheimer's disease, osteoporosis, lung disease, kidney disease, Parkinson's disease, and dementia. It is difficult for elderly people to take care of themselves in presence of the aforementioned clinical conditions. This leads to poor quality of life. Further, the diagnosis and monitoring of chronic diseases need continuous effort. In this regard, various IoT-driven devices have shown potential in dealing with these diseases. Several studies [81–85] in the past have proposed IoT-based solutions to improve the living standard of geriatric patients with the aforementioned diseases. Winkler et al. asserted that remote monitoring in geriatric care can reduce the mortality rate along with hospitalization rate and delivered quality chronic disease treatment [86]. In [84], the authors proposed a system to monitor vital signs that help to detect various chronic diseases of geriatric patients using a range of wearable sensors. Data mining approaches were adopted for training the system. In [87], a fuzzy ontology-based healthcare system (Figure 6) was employed to ensure continuous monitoring of diabetic patients. The system ensured the monitoring of diet status and health conditions. The device was able to provide recommendations for leading a healthy life. Demirl et al. have reported the application of IoT, which helped geriatric patients to deal with dementia [4]. Elderly people with dementia mostly show decreased mental and physical efficiency due to memory loss. The authors designed a system to collect, transmit, and record the data from various sensors that were placed in the patient's home (in the kitchen, bedroom, toilet, and bathroom). All routine behaviors of the patients were recorded, which were used to train the machine learning model. The system also alerted the patients when there was a deviation from the routine activity. Cardiovascular diseases (CVD) can be a potential reason for the high mortality rate among the geriatric population. CVD may further lead to other health issues such as angina, myocardial infarction, atrial fibrillation (AF), and heart attack. Hence, aging people with CVD need care and continuous monitoring. Electrocardiograph (ECG) signals reflect the functionality of the cardiac muscle and act as an indicator of various cardiac abnormalities. Hence, in most IoT-based healthcare systems, ECG has been a recording parameter of choice for researchers. In [88], the authors proposed a smartwatch (from Apple) with Kardia Band (KB) technology that can detect AF effectively using a single-lead ECG signal. The proposed device could effectively differentiate sinus rhythm from AF by comparing the cardiac expert interpreted ECG with KB recorded ECG. Such a device was paired with a mobile application for automated detection of AF. In another research [89], the authors proposed a portable IoT-driven ECG monitoring device (3 electrodes) by integrating a single-chip computer and various sensors. Herein, the acquired data was stored in the cloud, which was made accessible to authorized personnel only. A reminder e-mail was automatically sent to the patient and doctors in case of abnormal ECG. The proposed device was also capable of measuring other health parameters like temperature, BP, and blood sugar level. In a similar study [90], a multisensory device was designed, which collected heart rate, temperature, and body activity data from the patients. These data were used for the predictions of heart attack or cardiac arrest. The integrated system employed signal processing and machine learning algorithms for the prediction. In the clinical environment, the ECG was acquired using a 12-lead ECG system, where the signal was recorded using 10 electrodes. The electrodes were connected to specific locations on the body surface [91]. However, when it comes to continuous ECG monitoring, using a 12-lead system is not comfortable. Hence, the single-lead ECG signals were acquired using a smart device such as a smartwatch and smart fabric. These signals can be used to diagnose various heart-related abnormalities. Kardia Mobile 6L [92] is the first IoT-based personal 6-lead ECG recording system that has been approved by the Food and Drug Administration (FDA). This is a compact medical-grade personal ECG device, which has been integrated with a mobile application called “Kardia” to monitor cardiac activities. In the future, enzyme-based heart abnormality devices may be developed. Troponin is a cardiac enzyme that is released on the damage of heart muscles and can act as a measure for the early diagnosis of the acute coronary syndrome [93]. Unfortunately, there is no on-site testing device available for measuring troponin levels. The development of such a device could speed up the diagnosis process for clinicians and helps in saving a life. This could be explored in the future.

Chronic kidney disease (CKD) is a condition that impairs the excretory functions of the kidney. CKD causes a reduction in urine output and fluid imbalance of the body. Diabetes, hypertension, and other health issues are the leading cause of CKD. These associated health conditions are making the diagnosis of CKD a complex process. The application of IoT, along with other healthcare technologies, is helping the physician for easy and efficient diagnosis and monitoring. In [94], the authors proposed an IoT-based system for diagnosing CKD by measuring salt intake, water intake, activity level, and sleeping pattern. This health information was stored in the cloud and later can be accessed by the physicians who analyze these data for diagnosis. Hosseinzadeh et al. [95], in their study, have developed a predictive model using the IoT-multimedia database for CKD detection. Using the health information from the database, the performance measures of the predictive models were computed. Herein, the selection of information was based on the clinical observations made by the physicians. Despite the efficient use of technology, most hospitals still rely on the manual monitoring of the urine output of the patients using the urine bag, which may be susceptible to human error. Numerous researches have already been dedicated to designing an IoT-based system [9, 94] for measuring urine output. However, these developed systems are still lacking acceptance by health professionals. Some of the potential reasons behind this may be the lack of commercialization of these devices, validation before their application, and insufficient awareness of their use.

### 4.4. Monitoring Mental Health and Cognitive Diseases

The mental illness severely impacts the routine life and social-economic status of elderly people. Mental health declines with progressing age. This may be the potential reason behind the increased mental illness in the elderly population. The most commonly observed mental health issues in the elderly include Alzheimer's disease, Parkinson's disease, dementia, depression, and schizophrenia. Numerous studies [58, 96–98] have employed IoT-based technology in the detection of these conditions and provided better care. de la Torre Díez et al. have reviewed the contribution of IoT in dealing with mental illness. The authors have summarized how different IoT-based solutions had revolutionized the process of monitoring and diagnosis of these diseases [99]. In [100], the authors reported a system that can be used for tracking and monitoring mentally ill patients. In another study [101], the behavioral changes (e.g., sleeping pattern, repetitive action, and excess active level) in geriatric population have been assessed using various IoT-based sensors, which were employed to diagnose Alzheimer's conditions. In a similar study [102], an IoT-based system has been proposed to analyze the efficacy of the medication by monitoring the symptoms of Parkinson's disease. The device consisted of an electronic dosing machine, a smartphone, a bed, and wrist sensors. The proposed device was able to monitor motor function, physical exercise, medication compliance, and meal intake time. The authors concluded that the proposed system can assist mentally ill geriatric patients to have an insight into the correlation between medication and symptoms of the disease. Parkinson's disease causes a deterioration of functions and leads to slower movement and restricted activity. The conventional methods follow a “pull test” for the early diagnosis of Parkinson's disease. The test detects postural instability using the Unified Parkinson's Disease Rating Scale (UPDRS) [103]. The primary challenge in such an IoT-based system for diagnosing mental illness is the efficient handling of a large volume of data that is acquired from various sources. A large dataset, generated from these devices, needs extra effort for processing and analysis to provide smart healthcare services. It has also been reported that various psychiatric biomarkers such as proteins and other macromolecules have potential in detecting mental disorders [104, 105]. However, more research is needed to reach the stage where it can be used clinically. In the future, biosensors that can efficiently detect these biomarkers may be employed in IoT-based systems for the efficient detection of various mental disorders. The elimination of the aforementioned issues can provide a long-lasting solution to deal with mental health issues, especially in the case of the elderly.

### 4.5. Telerehabilitation

Rehabilitation plays an important role to counteract physical disability and impairment caused due to either aging or postillness (e.g., heart attack, falls, total hip, knee, and joints replacement) surgery. Numerous researches [41, 106] have employed sensor-integrated systems, wearable sensors, and virtual reality for providing rehabilitative services to geriatric patients. In [107], the authors have facilitated in-home rehabilitation of the geriatric patient through monitoring of the activity (still, up/down, walking, idle, running, and cycling) and movement (arm press, arm twist, arm circles, curls, and shoulder rolls) recognitions. These movements are monitored using a smartphone embedded with accelerometer sensors. Further, the prediction of these activities could be made using different machine learning algorithms. Nave and Postolache (2018) have designed an IoT-based smart walker rehabilitation system for geriatric patients. Such a system can monitor the walking matrics during a rehabilitation session. Various sensors such as ultrasound sensors, load cell sensors, and inertial management sensors have been included in this system. Herein, the recorded data was transmitted to the cloud using a smartphone [108]. A number of research articles have also been reported on monitoring postoperative rehabilitative therapy [107, 109, 110]. These studies have also proposed the concept of developing a smart home that is integrated with numerous embedded sensors (e.g., room occupancy, silhouette sensors, etc.) for the in-home monitoring of geriatric patients.

The most common cause for adopting telerehabilitation is to avoid a repeated visit to the physiotherapy center and its associated cost. In this regard, the acceptability of a rehabilitative device mostly depends on its costs. However, the current rehabilitative devices are having a high cost as compared to the cost of accessing physiotherapy. Hence, there is a need to develop low-cost rehabilitative devices in the future [111]. Although several IoT-based rehabilitation systems have been reported in the past for elderly people, rehabilitation for persons with disabilities has been ignored. This should be explored in the future. Easy accessibility of these devices is another major challenge for their acceptance. A user-friendly interface and improved accessibility in system design will help patients to complete the treatment process without much assistance from other individuals. Further, it will encourage the participants to stay with the rehabilitation program and encourage them to actively participate in the exercise. Most of the conventional rehabilitation systems restrict their outcome to the training results. However, the outcome measurement should also include the quality of practice in terms of speed, accuracy, kinematics, and daily home activities outside the training session [112]. The inclusion of such type of outcome measurement unit will enable the IoT-based rehabilitation system to qualitatively assess the patient's improvement and manage their exercise routine [113].

### 4.6. Monitoring Nutrition and Medication

Nutrition plays an important role in the overall well-being of a person. The deficiency of a healthy diet may lead to malnutrition. The possibility of malnutrition is comparatively high in the elderly population. Lack of timely diagnosis may later cause various health issues such as cardiovascular diseases, diabetes, and osteoporosis. Hence, it is necessary to track food habits and daily nutrition, especially for elderly people who are at higher risk of malnutrition. Lin et al. [114] have proposed a system for daily diet control of elderly persons using a single-chip computer (Raspberry Pi), two LEDs, and RFID cards. The system contains a list of foods that may cause various diseases (CVDs, degenerative diseases, and osteoporosis). Further, it recommends food to the patients based on their health reports. In a similar study [115], a device that can recommend daily nutritional diets and exercise for elderly people has been proposed. Apart from nutrition, various other factors influence the health of the elderly. Through proper nutrition, one can improve the health status, but it is not sufficient for people who are already dealing with one or more chronic diseases. Maintaining a routine medication regime is essential for them in addition to the diet. With the passing age, elderly people experience a decline in cognitive as well as mental ability. Hence, maintaining timely and proper medication is becoming challenging. A smart pillbox or pill dispenser can be a potential solution to the aforementioned issue, which can remind the patient of their medication. The IoT-integrated smart pillboxes have gained much popularity in the recent years. These devices enable the caregivers and doctors to remotely monitor and control the medication routine of the patients. An IoT-based smart pill dispenser has been proposed in [116] for monitoring the medication regime of geriatric patients. Herein, a mobile application has been used to alert the patients and caregivers in case of an incorrect medication schedule. In [113], the authors have proposed a reminder-cum-memory aid system that can assist geriatric patients with dementia. The proposed system could generate an e-mail, audiovisual display, and text message to remind the patients of their medication schedule.

Managing polypharmacy is a potential challenge during medication. The management of multiple drugs, maintaining their dose information, and medication time require efficient algorithms. Again, different medicines do not follow the same storage condition. Maintaining different storage environments for the pills in a single pill chamber is difficult. This issue can be solved by creating different subchambers in the pill tray, where the storage condition for each subtray can be maintained as per requirement. It may be possible that some users may accidentally consume the wrong medicine either due to their forgetfulness or due to any system error that may suggest the intake of an incorrect medicine. For these issues, the IoT-based smart medication system may include a feature that will provide the details of the pills to be consumed to the user. Most of the IoT-based medication systems focus only on giving the correct medicine at the right time to the patients. However, these systems have ignored the requirement of the “pill-restocking alerts” system. Inclusion of the pill-restocking information will remind the family members as well as the caregivers to refill the medicine before time so that the medication regime of the patients will not be hampered. This may be incorporated in the future. Also, in the future, the medication system must include multiple reminder signals such as voice, text, vibration, and sights (as in [113]) which can help elderly people with impairment. Such devices will increase their acceptability.

### 4.7. Emergency Healthcare Service

Emergency healthcare services deal with various sudden and unpredicted health crises such as accidents, falls, and heart attacks. Aging causes functional impairment and forgetfulness that make these elderly people prone to a sudden health crisis. Emergency care services are an integral part of every care service and can be employed while monitoring either health parameters, human physical, and behavioral activities or falls and so forth. In the past, the emergency service was only accessible in the hospital under the direct supervision of the health professional. However, with the rapid growth in technology and Internet services, it is now possible to avail of these services at home. Numerous studies [114, 117, 118] have been reported in the past for remote monitoring of daily activities through IoT-based systems. An IoT-based living assistance system has been proposed in [119], which can monitor and register patient's vital information. The system also poses a triggering system in the case of an emergency. In [24], an IoT-aware health monitoring system has been implemented, which sends alerts to health professionals when elderly people require either hospitalization or emergency medical attention. An emergency system has been integrated with the telemedicine system in [120]. Herein, the system shows the user location as well as emergency information to the caregivers and also provides instructions to help the patient. Korzun et al. [121] proposed digital assistance services for emergencies. In case of an adverse medical situation, when the patients feel the need for emergency care, a signal can be sent to their relatives with a single button press. However, the emergency message is automatically generated and sent to the caregiver in case of a fall event [122].

The current IoT-based emergency healthcare monitoring system for elderly care only focuses on handling a single medical condition of elderly people. Since a medical emergency may arise from a wide array of health conditions, monitoring a single health parameter would be insufficient to achieve an efficient healthcare service. In [109], the authors reported that there is a need for an integrated emergency system that will employ more sensors (like blood pressure meter and heart rate meter) and new services to diversify the healthcare features and benefits. Immediate and fast communication is crucial while dealing with an emergency. Elderly people need to contact either a family member or the caregivers to get swift support. Most of the IoT-based emergency systems use the Internet for the communication purpose, where a network disconnectivity may fail the whole system. Hence, in the future, these systems must include an alternate mode of communication which will be activated at the time of network disconnectivity.

## 5. Current Issues, Challenges, and Future Scope

The development of an efficient healthcare device/system for the geriatric population must be based on three important aspects, a fast response time during first-aid service, an effective communication system, and a user-friendly interface, for its efficient use. Lacking a user-friendly interface may lower the efficiency of the system. The current healthcare devices are incapable of providing fast access and a valid communication system. Hence, it is inevitable to develop futuristic devices that overcome these issues and provide more reliable solutions. An ample amount of research in the literature is focused on IoT-based healthcare applications for geriatric patients. IoT technologies have significantly transformed healthcare services and have provided novel solutions to various healthcare problems. Despite its wider applications, many challenges still exist in this research domain. Most of the currently available solutions for elderly care have ignored the functional limitations associated with age and cognitive performance. The types and modes of disabilities in old age are person-specific and demand the development of adaptive systems. The recently developed IoT-based systems are relatively user-friendly as compared to the devices developed in the past. However, these systems have ignored the fact that, as compared to younger people, elderly people are slow learners. Moreover, elderly people face difficulty in operating advanced gadgets and take a longer time to adapt to a new system. One potential solution for this aforementioned issue is to consider the comprehensive knowledge on behavioral analysis as a feedback mechanism while designing the IoT-based healthcare system.

A large number of vendors who provide a range of products, protocols, and devices are present in the healthcare industry. However, there is no compulsion on these vendors to follow specific rules and regulations. Due to the variations in the communication protocols, standards, and design, various interoperability issues may arise. Hence, standardization of these aforementioned areas is crucial. The employment of standardization to these medical devices may provide fundamental protection and support robust security solutions for software development. Two examples of such standardization agencies are Systematized Nomenclature of Medicine (SNOMED) and Logical Observation Identifiers Names and Codes (LOINC). Although the advancement in the IoT healthcare applications makes the process of standardization more complex, no dedicated study was found in the literature which had addressed these issues and demands future attention. IoT-based healthcare network connects a large number of medical devices and sensors generating a higher volume of medical data. These data are distributed across various data sources/repositories. Hence, to avoid complexity in the data flow among various sources, a suitable method for data integration is required. Since healthcare data contains private and sensitive information of the patients, the privacy and security of data can be a major issue that needs to be handled with caution and using suitable protocols. Currently, there are no standard protocols and regulations available which will control the way the health information is collected in an IoT-embedded system [123]. Hence, the use of an efficient security and privacy safety protocol is the primary requisite during data transmission. Personalized healthcare services include the involvement of multiple doctors and caregivers from different geographical areas. This requires smooth communication among these service providers. For this purpose, the future healthcare system would require a data model that can not only identify and integrate various data sources but also provide an efficient data-handling mechanism. The IoT-based solutions capture personal data about the location, health parameters, and daily habits during monitoring. These data were then stored in a cloud server that can be accessed and shared by third-party solution providers. The cloud layer acts as a repository of the details, medical history, and other physiological parameters of the patients. Unfortunately, the extraction of healthcare data from the cloud reveals the personal information of the patients. This may hamper the privacy of elderly people who usually stay alone at home and may put them at a higher risk category. Hence, there should be a provision for selective sharing of the data. Further, the system may take the consent of the patients before using their health information. Data encryption can be a potential solution to the privacy issue that can reduce information security risks and should be explored in the future.

Despite various technological and design advancements in IoT-based systems, evaluating usability and acceptability is still a major challenge. The measurement of willingness to use and keep, simplicity, patient satisfaction level, reliability, and wearable time are some of the measures to assess usability and acceptability. To make an IoT-based healthcare system more popular, modifications in manufacturing are required to address some critical issues such as power consumption, restricting the user movement within a confined area, and cost [124]. The cost of a device is directly related to its acceptability. Hence, more research must be dedicated in the future to develop low-cost healthcare devices. This can be achieved using low-cost materials and cheaper sensors in future development. A cost analysis can be performed to have an overall idea of the cost of the commercially available devices developed so far. This will help geriatric patients to opt for accurate and cost-effective solutions to their health problems.

## 6. Conclusion

The substantial growth in the aging population in the recent years has caused various health and socioeconomic challenges in the day-to-day life of geriatric people. Numerous technological advancements in the healthcare sector have addressed these challenges. They have also contributed to the development of various solutions associated with the underlying problems and help them to live a normal life. The current study discussed the need for IoT-based systems in addressing the health issues associated with geriatric persons. Further, the paper also discussed the role of IoT in influencing various healthcare domains (AAL, telemedicine, robotic technology, wearable sensors, etc.) in the elderly population. Up-to-date information regarding the current status of the IoT-based healthcare systems that deal with various healthcare issues such as chronic diseases, mental illness, cognitive diseases, and medication adherence is discussed in the paper. Finally, various issues and potential limitations of the existing healthcare systems/devices are also mentioned. The review paper will help future researchers to have a piece of comprehensive knowledge in the aforementioned field, which they can analyze to understand the gaps in the current research and subsequently use this information to develop advanced and intelligent healthcare systems.

## Data Availability

No data were used to support this study.

## Supplementary Materials

Supplementary Materials The supplementary file S1 contains information regarding the basic architecture of an IoT-based system used for the health monitoring of the geriatric population. The section describes the three basic layers of the IoT system architecture.
