# Supplementary material for: The Internet of Things in Geriatric Healthcare
Source: J Healthc Eng. 2021 Jul 17;2021:6611366. doi: 10.1155/2021/6611366 (PMC8313366; doi:10.1155/2021/6611366)
Supplement: Supplementary Materials — The supplementary file S1 contains information regarding the basic architecture of an IoT-based system used for the health monitoring of the geriatric population. The section describes the three basic layers of the IoT system architecture. [file 6611366.f1.docx]

**Supplementary Information:**

**S1. Basic architecture of IoT-based system**

The IoT-based system is an integration of several distinct elements (e.g. sensors, actuators, network protocols, and cloud services), which are organized according to system architecture that helps to generate a meaningful result. Such a system offers features like data collection, transmission, and data analysis [1]. Further, it provides uninterrupted connectivity among objects to exchange, collect, and combine data through a remote server. According to the features, functionalities, and specifications, the system architecture is represented in three layers, namely, the perception layer, gateway layer, and cloud layer [2, 3]. However, the layer structure may change either based on the application or as per user perspective. Figure 1 shows a basic architecture for remote health monitoring of geriatric patients. Each layer of this system is described in this section.


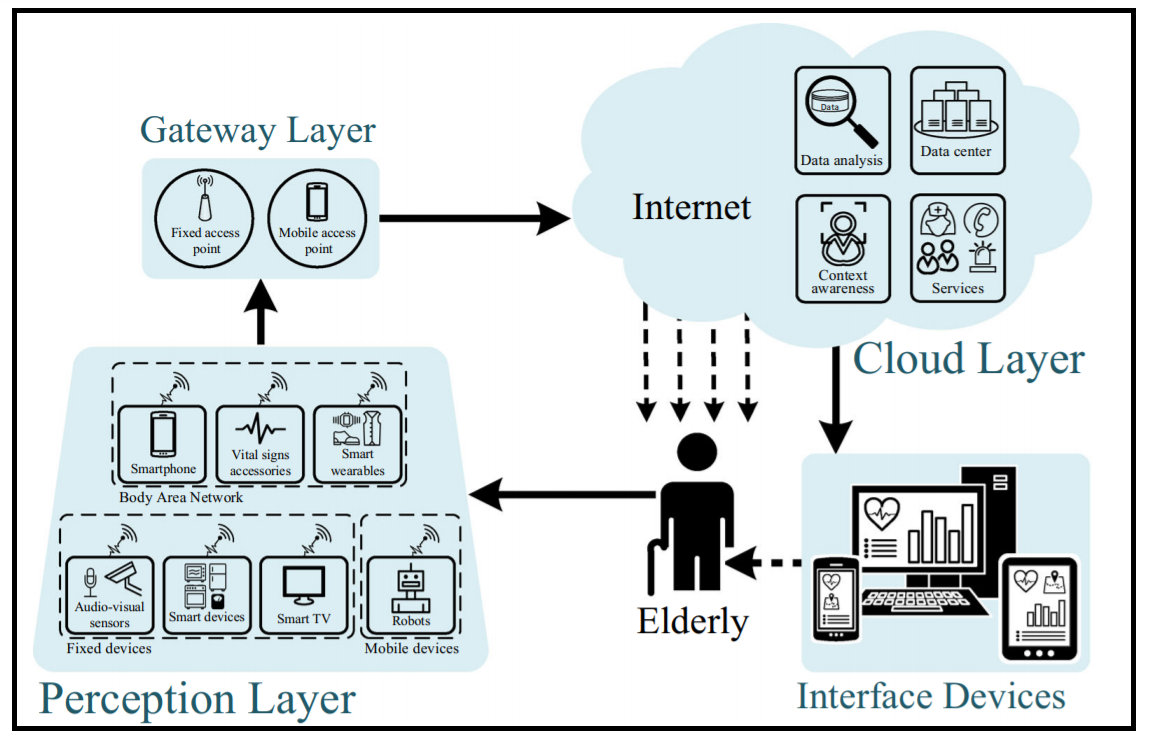


Figure 1: The basic architecture of IoT driven remote monitoring of geriatric system (Reproduced from [4])

**S1.1. Perception layer**

The perception layer is the first layer of the proposed architecture that acts as an interface between the system and the user. This layer uses a variety of sensors, actuators, and medical devices devoted to capturing the vital health information and environmental data of the person under monitoring. The recorded information is converted to a digital domain and then transmitted to the higher layer for further processing [4]. Medical devices such as electrocardiography devices, heart rate monitoring devices, pulse oximeter, blood pressure monitoring devices, etc. are used for continuous monitoring of patients. Additionally, IoT-driven smart devices like smart hats, smartwatches, and fitness trackers are used in this layer for obtaining the patients’ health status. Health parameters like heart rate, blood pressure, respiration rate, blood glucose level, body temperature, etc. are collected using such devices.

**S1.2. Gateway layer**

The gateway layer is the middle layer of the IoT system. This layer enables the sensors to get connected to a remote server via a wireless network. The wireless network uses different transmission media that include Bluetooth (IEEE 802.15.1), Zigbee (IEEE 802.15.4), Wideband Code Division Multiple Access (WCDMA), Wireless Local Area Networks (WLANs) (IEEE 802.11 variants), and Worldwide Interoperability for Microwave Access (WiMAX) (IEEE 802.16) as per application [5]. This layer receives data from the perception layer and uses the above-mentioned technology to transmits the gathered data to the Cloud layer for further processing through the local gateway. The gateway helps to provide continuous connectivity to various sensors in the perception layer and manage the interruptions in connectivity.

**S1.3. Cloud layer**

The cloud layer is the third layer of the IoT system. As illustrated in fig. 1, this layer was present in the data center which is usually placed in a remote location. The data collected from the gateway layer of the system was stored in the data center where further processing and analysis was done by the cloud layer. The cloud can be designed in two ways. First, through the interconnection of the local hospital information system (HIS) and the local server. This approach provides more protection in terms of privacy and security. Second, through an internet-connected third-party remote server [6]. The gathered data get processed using the high processing power of the cloud platform with the help of various data analysis techniques such as data fusion, data analytics, machine learning algorithm, and data reasoning to obtain innovative ideas and results regarding the stored data. These back-end technologies could assist physicians in the diagnosis of various chronic diseases like the prediction of blood glucose concertation in diabetes patients, high blood pressure, depression, and acute diseases like the prediction of heart attack, asthma attack, pneumonia, etc. [7, 8]. Further, it can also predict behavioural change like mild cognitive impairment in the geriatric population [9]. Afterward, based on the obtained results, decisions are made and provided to the respective physicians and geriatric patients. This system empowers the caregiver and healthcare personnel to take appropriate decisions against various unpredicted catastrophic events in the geriatric population.

Reference:

[1] L. Atzori, A. Iera, and G. Morabito, "The internet of things: A survey," *Computer networks,* vol. 54, no. 15, pp. 2787-2805, 2010.

[2] F. Touati and R. Tabish, "U-healthcare system: State-of-the-art review and challenges," *Journal of medical systems,* vol. 37, no. 3, pp. 1-20, 2013.

[3] A. Al-Fuqaha, M. Guizani, M. Mohammadi, M. Aledhari, and M. Ayyash, "Internet of things: A survey on enabling technologies, protocols, and applications," *IEEE communications surveys & tutorials,* vol. 17, no. 4, pp. 2347-2376, 2015.

[4] I. Azimi, A. M. Rahmani, P. Liljeberg, and H. Tenhunen, "Internet of things for remote elderly monitoring: a study from user-centered perspective," *Journal of Ambient Intelligence and Humanized Computing,* vol. 8, no. 2, pp. 273-289, 2017.

[5] N. Hussien, I. Ajlan, M. M. Firdhous, and H. Alrikabi, "Smart Shopping System with RFID Technology Based on Internet of Things," 2020.

[6] R. Mieronkoski *et al.*, "The Internet of Things for basic nursing care—A scoping review," *International journal of nursing studies,* vol. 69, pp. 78-90, 2017.

[7] C. Zecchin, A. Facchinetti, G. Sparacino, and C. Cobelli, "Jump neural network for online short-time prediction of blood glucose from continuous monitoring sensors and meal information," *Computer methods and programs in biomedicine,* vol. 113, no. 1, pp. 144-152, 2014.

[8] N. Liu *et al.*, "An intelligent scoring system and its application to cardiac arrest prediction," *IEEE Transactions on Information Technology in Biomedicine,* vol. 16, no. 6, pp. 1324-1331, 2012.

[9] A. Akl, B. Taati, and A. Mihailidis, "Autonomous unobtrusive detection of mild cognitive impairment in older adults," *IEEE transactions on biomedical engineering,* vol. 62, no. 5, pp. 1383-1394, 2015.
